# Supplementary material for: Facile Approach to the Fabrication of Highly Selective CuCl-Impregnated θ-Al2O3 Adsorbent for Enhanced CO Performance
Source: Materials (Basel). 2022 Sep 13;15(18):6356. doi: 10.3390/ma15186356 (PMC9504217; doi:10.3390/ma15186356)
Supplement: Supplementary file 1 [file materials-15-06356-s001.zip › materials-1856246-supplementary.pdf]

Supplementary Material

Facile Approach to the Fabrication of Highly Selective CuCl-Impregnated θ-Al_2O_3 Adsorbent for Enhanced CO Performance

Cheonwoo Jeong[1,*], Joonwoo Kim[1], Joon Hyun Baik[2,3], Sadanand Pandey[4] and Dong Jun Koh[1,*]

[1]*Particulate Matter Research Center, Research Institute of Industrial Science & Technology (RIST), 187-12, Geumho-ro, Gwangyang-si, Jeollanam-do, 57801, Republic of Korea*

[2]*Department of Chemical and Biological Engineering, Sookmyung Women's University, 100 Cheongpa-ro 47-gil, Yongsan-gu, Seoul 04310, Republic of Korea*

[3]*Institute of Advanced Materials and Systems, Sookmyung Women's University, 100 Cheongpa-ro 47-gil, Yongsan-gu, Seoul 04310, Republic of Korea*

[4]*Department of Chemistry, College of Natural Sciences, Yeungnam University, Gyeongsan, Gyeongbuk, 38541, Republic of Korea*

Correspondence: cjeong@rist.re.kr (C.J.); djkoh@rist.re.kr (D.J.K., co-correspondence)

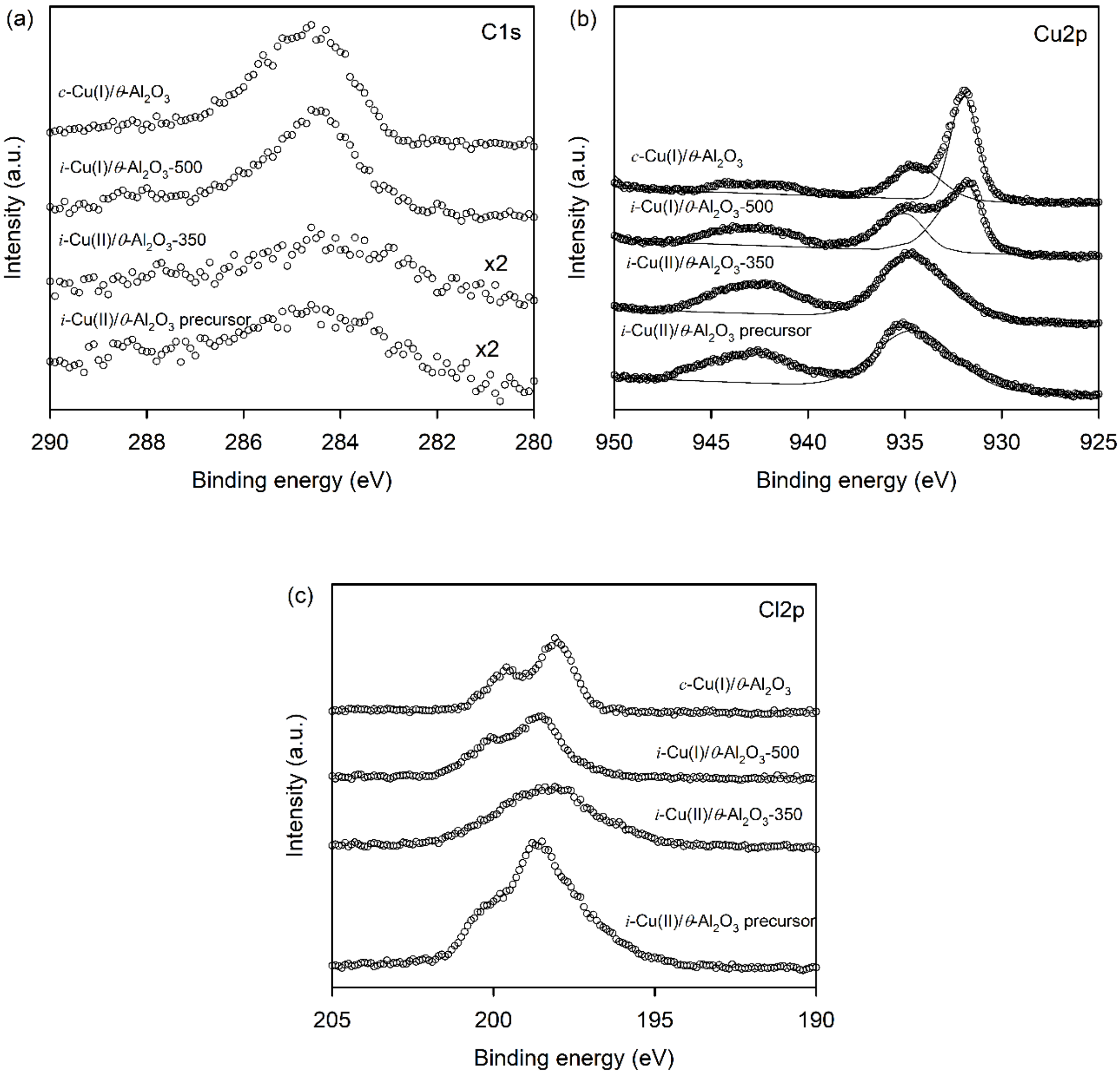

Figure S1. XPS spectrum of (a) C1s, (b) Cu2p and (c) Cl2p of *i*-Cu(II)/*θ*-Al_2O_3 precursor, *i*-Cu(I)/*θ*-Al_2O_3-350, *i*-Cu(I)/*θ*-Al_2O_3-500 and *c*-Cu(I)/*θ*-Al_2O_3. XPS measurement was carried out on an ESCA LAB250 (VG scientific) with Al Kα source (1486.6 eV). The power applied to the X-ray gun was 15 mA and 15 kV, and the step size of scan was 0.1 eV.

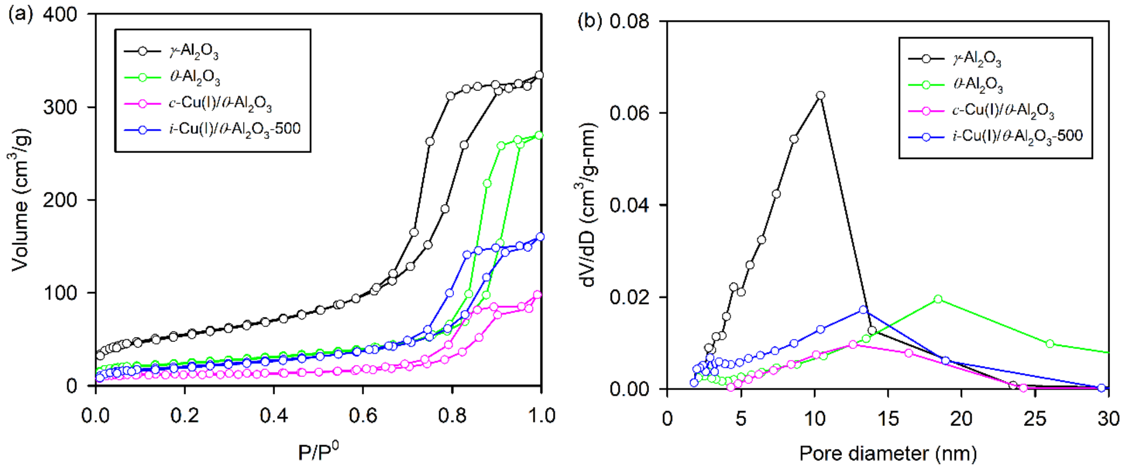

Figure S2. (a) N_2 adsorption and desorption isotherms and (b) pore size distribution plot of *γ*-Al_2O_3, *θ*-Al_2O_3, *c*-Cu(I)/*θ*-Al_2O_3 and *i*-Cu(I)/*θ*-Al_2O_3-500. N_2 adsorption isotherms were carried out at 77 K in liquid N_2 on the Micromeritics ASAP 2010. The samples were pretreated at 200 °C in vacuum for 3 h before measurement.

Table S1. BET surface area, pore volume and average pore diameter of *γ*-Al_2O_3, *θ*-Al_2O_3, *c*-Cu(I)/*θ*-Al_2O_3 and *i*-Cu(I)/*θ*-Al_2O_3-500.

Samples	S_{BET} (m^2/g)	V_{Total} (cm^3/g)	d_{Avg} (nm)
γ-Al_2O_3	191	0.51	8.9
θ-Al_2O_3	86	0.41	17.3
c-Cu(I)/*θ*-Al_2O_3	50	0.15	14.7
i-Cu(I)/*θ*-Al_2O_3-500	72	0.24	10.8

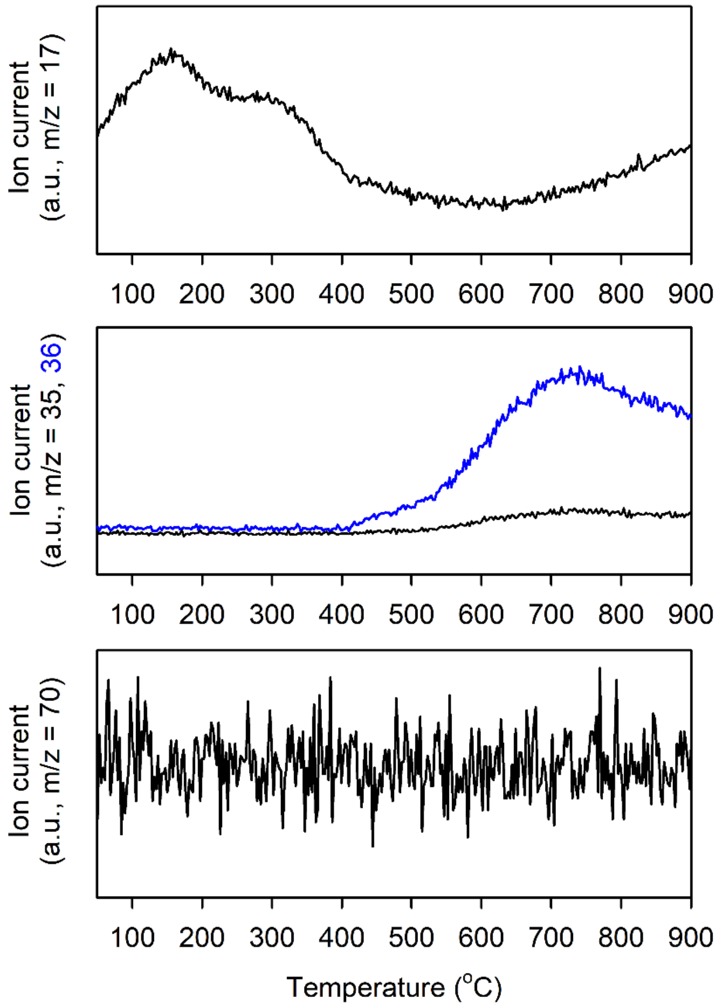

Figure S3. TG-MS curves of *i*-Cu(II)/θ-Al_2O_3 precursor. Mass spectrum was measured by HIDEN-20 R&D mass spectrometer.
